# Multidrug-resistant *Acinetobacter baumannii* outbreaks: a global problem in healthcare settings

**DOI:** 10.1590/0037-8682-0248-2020

**Published:** 2020-11-06

**Authors:** Mariana Neri Lucas Kurihara, Romário Oliveira de Sales, Késia Esther da Silva, Wirlaine Glauce Maciel, Simone Simionatto

**Affiliations:** 1 Universidade Federal da Grande Dourados, Laboratório de Pesquisa em Ciências da Saúde, Dourados, MS, Brasil.

**Keywords:** Risk factors, Multidrug-resistant, ICU

## Abstract

**INTRODUCTION:**

The increase in the prevalence of multidrug-resistant *Acinetobacter baumannii* infections in hospital settings has rapidly emerged worldwide as a serious health problem.

**METHODS:**

This review synthetizes the epidemiology of multidrug-resistant *A. baumannii*, highlighting resistance mechanisms.

**CONCLUSIONS:**

Understanding the genetic mechanisms of resistance as well as the associated risk factors is critical to develop and implement adequate measures to control and prevent acquisition of nosocomial infections, especially in an intensive care unit setting.

## METHODS

A comprehensive search of the literature was performed using PubMed, ScienceDirect, and Web of Science. The search was restricted to original articles published in English related to risk factors, epidemiology, and multidrug-resistant *A. baumannii* (MDR-*Ab*). The key words used were (*Acinetobacter baumannii* OR *A. baumannii)* AND infection AND (multidrug-resistant OR MDR) AND (ICU), or (*Acinetobacter baumannii* OR *A. baumannii)* AND risk factors AND epidemiology. Case reports or conference abstracts were excluded. Two independent investigators searched the electronic databases using an identical method. The full texts of articles were reviewed by two independent reviewers to determine whether they met the eligibility criteria for inclusion. References in the included articles were reviewed to explore additional papers.

## *ACINETOBACTER BAUMANNII* CONTEXT

*Acinetobacter* spp. is a pathogen that belongs to the *Moraxellaceae* family, which consists of 59 different species^1^ ^,^ ^2^. In this family, *Acinetobacter spp.* is the fifth most frequently isolated microorganism, distributed across five continents, among the gram-negative bacteria involved in nosocomial infections^3^. It is known that the species *Acinetobacter baumannii* is an opportunistic pathogen with clinical relevance^3^ ^-^ ^6^. The most frequent clinical manifestations are pneumonia associated with mechanical ventilation, bloodstream infections, urinary tract infections, and bacteremia associated with long periods of device use, meningitis, eye infections, intra-abdominal infections, surgical sites, the respiratory tract, and the gastrointestinal tract^7^ ^,^ ^8^. Nonetheless, this pathogen can survive in the intensive care unit (ICU) environment for up to four weeks due to its capacity to produce biofilms and thus contaminates patients admitted later^9^. Lipopolysaccharides (LPS), vesicles and proteins, polysaccharide capsules, phospholipases, proteases, outer membrane porins, and iron uptake systems are the most important factors for *A. baumannii* resistance^10^.

MDR-*Ab* is considered a hospital-acquired infection, which has been rapidly increasing worldwide due to the fitness effect of its resistance mutations^3^. The exacerbated and undue use of antibiotics associated with ineffective hospital interventions are related to the spread of MDR and consequently reduce treatment options. The World Health Organization (WHO) published in early 2017 a list of priorities for research into the development of active antibiotics against MDR and extensively resistant bacteria, which put *A. baumannii* first in the list of critical situations around the world^11^. It was estimated that multidrug-resistant *A. baumannii* can cost $33,510 to $129,917 per infection^12^. Moreover, patients with bacteremia can be related to high mortality rates due to multidrug-resistant *A. baumannii* (56.2%), when compared to *A. baumannii* strains with no multidrug resistance *(*4.7%)^13^. An average of 10.6% of patients die as a result of infections caused by MDR-*Ab* ^12^.

## OVERVIEW OF *A. BAUMANNII* ANTIBIOTIC RESISTANCE

The key resistance mechanisms of *A. baumannii* are the low permeability of the outer membrane, alteration in antibiotic binding sites, and mutations, which can cause upregulation or downregulation of efflux system activity^4^ ^,^ ^10^. Among these mechanisms, alteration of bacterial membrane permeability by the outer membrane proteins (OMPs) is associated with the loss or reduced expression of porins^8^. This group is represented by OmpA, OprD, and CarO proteins^14^. The OccD1 (OprD) channel of the *Pseudomonas aeruginosa* species plays an important role in the uptake of molecules such as imipenem and meropenem. This OM channel is closely related to the OM family in *A. baumannii* and is the largest pore described amongst Occ proteins with efficient *in vitro* uptake responsible for transporting small molecules, presenting a huge potential for future antibiotic design^15^.

The efflux system expels toxic compounds to the extracellular environment. Within it, five families of systems have been described in *A. baumannii*, such as the major facilitator super family (MFS), ATP binding cassette (ABC), resistance nodulation division (RND), small multidrug resistance family 1 (SMR), multidrug and toxic compound extrusion (MATE), and drug/metabolite transporter (DMT)^16^. The RND family is well characterized and is represented by the AdeABC, AdeIJK, and AdeFGH efflux system^17^. Mutations can influence the expression of the efflux system, resulting in increased cases of clinical infections. A study highlighted resistance to aminoglicosides, tetracyclines, chloramphenicol, fluoroquinolones, some beta-lactams, and tigecycline related to mutations on the chromosome or plasmids^18^. The efflux systems CraA, AmvA/AedF, Tet(A), and Tet(B) of the MFS system are known to have a drug-specific substrate profile, and are involved in chloramphenicol, erythromycin, chlorhexidine, and tetracycline resistance^19^ ^,^ ^20^. The expression of Acel protein is strictly related to chlorhexidine transportation and the *AbeM* gene (a member of the MATE family), which confers resistance to fluoroquinolones through the H^+^ antiport^20^ ^,^ ^21^. Quinolone resistance can be related to the *AbaQ* gene, which belongs to the MFS transporter and has its N- and C- ends located in the cytoplasm, which confers its characteristic as a drug H^+^ antiporter-1 (DHA1). *AbaQ* knockout in *A. baumannii* confirmed its involvement with quinolone susceptibility, resulting in decreased susceptibility caused by active efflux transportation^22^.

It is known that the fluoroquinolone resistance mechanism is mainly encoded by mutations in DNA gyrase (*gyrA, gyrB* genes) and topoisomerase IV (*parC, parE* ), with *gyrB* and *parE* mutated at a lower frequency. These mutations are sequential, as primary mutations in *gyrA81* are followed by mutations in *parC88* and *parC84* in *A. baumannii.* However, a study described strains carrying mutations in only the *parC* gene, revealing the involvement of other resistance mechanisms for fluoroquinolone^23^ ^-^ ^24^.

One of the main mechanisms of resistance to beta-lactam antibiotics is associated with changes in the structure or expression profile of penicillin binding proteins (PBPs)^25^. PBPs are transglycosylases, transpeptidases, and carboxypeptidases, enzymes located in the plasma membrane, and are involved in the synthesis of peptidoglycan, an essential component of the bacterial cell wall. Once a PBP is acylated by a beta-lactam antibiotic, it is unable to catalyze hydrolysis of the covalent acyl-enzyme intermediate and is inactivated. Peptidoglycan transpeptidation cannot occur; thus, the cell wall is weakened^25^.

PBPs are divided into high molecular mass (HMM) and low molecular mass (LMM). The first is responsible for insertion into the cell wall, which, depending on the structure and catalytic activity of the N-terminal domain, can be classified as class A or B^26^. Therefore, changes in PBP expression lead to decreased susceptibility to these antimicrobial agents, favoring the occurrence of beta-lactam-resistant strains^27^. Due to the lack of interaction that occurs in the connection between beta-lactams and PBPs, the susceptibility of *A. baumannii* strains to beta-lactams has been observed^27^ ^-^ ^29^.

Mutations can occur and modify the binding of antibiotics, inactivating some lipids, such as lipid A^30^. Polymyxins interact with lipid A through the addition of phosphoethanolamine (PEtn), resulting in displacement of cations Mg^2+^ and Ca ^2+^, which destabilizes the membrane. These molecules are mediated by the pmrCAB operon^31^ ^-^ ^33^. Alterations in the pmrA-pmrB two-component system, which is also involved in lipid A biosynthesis, upregulate pmrC, influencing the synthesis of PEtn. It is known that LPS is synthesized through the lpx pathway; mutations in *lpxA*, *lpxC*, and *lpxD* genes lead to deficiency in LPS production and its complete loss, conferring the colistin resistance phenotype^34^ ^-^ ^35^. Colistin resistance can be chromosomal or plasmid-encoded, carrying the *mcr* gene (*mcr-1* to *mcr-5*)^36^ ^-^ ^37^.

Carbapenemases, belonging to class A of Ambler (1980) and to group 2 of Bush and Jacob (2010) are considered one of the most versatile enzymatic families among β-lactamases, since they are able to hydrolyze most β-lactam antibiotics, such as carbapenems, penicillins, cephalosporins, and monobactams, in addition to being resistant against some commercial β-lactamase inhibitors^35^ ^-^ ^38^. Enzymes such as KPC-2, KPC-3, KPC-4, and KPC-10^39^, as well as GES-11, GES-12, and GES-14^40^, have already been described in *A. baumannii* ^38^.

Metallo-β-lactamases belong to class B of Ambler (1980) and group 3 of Bush and Jacoby (2010). They confer resistance against penicillins, cephalosporins, and carbapenems, and are inhibited by β-lactamase inhibitors (clavulanic acid, sulbactam, and tazobactam). The enzymes representing this family are VIM-1 and NDM-1, commonly related to penicillin hydrolysis^39^ ^-^ ^43^. Class C of Ambler (1980), group 1 of Bush and Jacob (2010), is represented by chromosomal cephalosporinases (AmpC), which hydrolyze penicillins, and cephalosporins at a low level. When the insertion element *ISAba1* or *ISAba125* is inserted upstream of the *bla*_AmpC_ gene, it is overexpressed, resulting in resistance to extended-spectrum cephalosporins as upstream *ISAba* induces strong promoter sequences^44^ ^-^ ^45^.

Oxacillinases belong to class D of Ambler (1980) and group 2 of Bush and Jacob (2010) and are encoded by the *bla*_OXA_ genes. These proteins hydrolyze carbapenems and penicillins at a low level and has weak hydrolysis of second and third generation cephalosporins^44^. Oxacillinases have been reported in clinical isolates of *A. baumannii* associated with hospital outbreaks^46^. Six subgroups of Class D carbapenem-hydrolyzing enzymes (CHDLs), including OXA-23, OXA-24, OXA-51, OXA-58, OXA-143, and OXA-235, were identified^47^. These enzymatic groups hydrolyze penicillins at a high level and carbapenems at a low level. However, the presence of insertion sequence (IS) is considered a strong promoter for the increase of oxacillin expression and dissemination^48^. It was reported that the *ISAba1/bla*_OXA-23_ or *ISAba1/bla*_OXA-51_ combination amplified resistance to carbapenems^49^.

Aminoglycosides bind to 16S rRNA in the 30S ribosomal subunits and inhibit protein synthesis. Resistance is mediated by aminoglycoside-modifying enzymes (AMEs), such as acetyltransferases (AAC), adenyltransferases (ANT), and phosphotransferases (APH), which are found on mobile elements such as transposons and plasmids. AAC enzymes are responsible for modifying amino groups, while the ANT and APH enzymes act on hydroxyl groups, breaking bonds and inactivating the antibiotic molecule^10^. Methylase production (*armA*, *rmtA*, *rmtB*, *rmtC*, *rmtD*) decreases the affinity of the aminoglycosides for 30S ribosomal subunits^50^. A study with carbapenem-resistant (CR) *A*. *baumannii* identified 97.2% of the isolates carrying the *aph(3´)-VI* gene, with the majority found in 4 different clusters (A, B, C, and E), conferring resistance to amikacin, and group D, harboring AME genes (*aac(6´)-Ib*, *aac(3)-Ia,* and *aph(3´)-Ia*), responsible for gentamicin resistance and intermediate resistance to amikacin^51^ ^,^ ^52^. The presence of methylase *armA* coexisting with *bla*_OXA-23_ in MDR *A. baumannii* has been previously described and identified in quinolone-resistant *A. baumannii* ^53^ ^,^ ^54^.

In addition to the multiple mechanisms of resistance, *A. baumannii* can acquire resistance genes through mobile genetic elements. Mobile elements, such as IS, transposons, genomic islands, integrons, and plasmids, are related to variations in the insertion site and carry strong transcriptional promoters that are abundantly synthesized^55^ ^,^ ^56^. Multiple *A. baumannii* plasmids have been reported: pA297-1, carrying gentamicin, kanamycin, and tobramycin resistance genes; pA297-3, carrying sulfonamide and streptomycin resistance genes; and pAb-G7-2, carrying an amikacin resistance gene^57^ ^,^ ^58^.

Transposons, such as *Tn2006, Tn2007*, and *Tn2008,* increase the spread of resistance genes and may present integrons, which were captured and express exogenous resistance genes^40^ ^,^ ^48^ ^,^ ^59^. Thus, integrons are composed of gene cassettes, and classes 1 and 2 are commonly found in *A. baumannii* clinical isolates^60^ ^-^ ^62^. As previously stated, insertion sequences act as strong promoters that increase the resistance levels of OXA carbapenemases in *A. baumannii* isolates^47^ ^,^ ^59^ ^,^ ^63^. Insertion sequence *Acinetobacter baumannii* (*ISAba)* can be located upstream of the resistant gene, overexpressing genes such as *AmpC* and OXA-51, which increases cephalosporin resistance^64^ ^,^ ^65^. Resistance to colistin in *A. baumannii* clinical isolates was related to the presence of the *ISAba125* at the 3' end of the *hns* gene, disrupting the normal expression of a transcriptional gene regulator^66^.

## RISK FACTORS RELATED TO *A. BAUMANNII*

Risk factors are directly related to increased susceptibility in hospitalized patients who develop some type of infectious disease involving bacterial resistance, consequently resulting in mortality in nosocomial environments. Investigation of the risk factors associated with *A. baumannii* infection/colonization contributes to the prevention and control of bacterial resistance, reducing the impact of *A. baumannii* isolates^67^ (Table 1 and Table 2). The prevalence of *A. baumannii* infection and colonization is higher in ICUs, since patients with severe clinical conditions are hospitalized in such wards. In addition, these patients have compromised immune systems due to the presence of comorbidities, altered nutritional status, prolonged hospitalization, invasive procedures, immunosuppressive drugs, and broad-spectrum antibiotics^67^ ^,^ ^68^.

**TABLE 1:** Risk factors associated with infection and colonization caused by *A. baumannii* in adult ICUs.

Study	Place of Study	Study Period	No. of Patients	Cases	Controls	Risk Factors	P-value
JANG et al., 2009	Taiwan	1997-2006	154	77 patients with *AB* bloodstream infection.	77 patients with bloodstream infection without *AB*.	Use of central venous catheter, mechanical ventilation, colonization by *AB*, respiratory failure, cardiovascular failure.	P < 0.05
YE et al., 2010	Germany	2001-2005	209	49 patients with MDR*AB*.	160 patients with CS*AB*.	Previous use of antibiotics, use of mechanical ventilation, > 60 years, length of hospital stay.	P < 0.05
ROCHA et al., 2008	Brazil	2005-2006	275	84 patients with PAVM.	191 patients without PAVM.	Stay > 7 days in hospital, use of corticoids, invasive procedures, use of central venous catheter, and tracheostomy.	P < 0.05
BROTFAIN et al, 2016	Israel	2005-2011	129	46 patients with pneumonia and positive sputum culture for MDR*AB* 72 h after MV onset and bacteremia.	83 patients with pneumonia and positive sputum culture for MDR*AB* 72 h after the onset of MV, without developing bacteremia.	Hospitalization > 3 days in the ICU, advanced age, and recent bacteremia.	P < 0.05
BLANCO et al., 2017	United States	2005-2009	101	90 patients with MDR*AB*.	11 patients with CS*AB*.	Advanced age, previous hospitalization, heart failure, paralysis, HIV-AIDS, and rheumatoid arthritis.	P < 0.05
ELLIS et al., 2015	United States	2006-2012	671	302 patients with infection caused by MDR*AB*.	369 patients with infection caused by CS*AB*.	Length of hospital stay, transfer from another hospital, previous use of antibiotics	P < 0.25
HENIG et al., 2015	Israel	2007-2012	2380	1190 patients with CR*AB*.	1190 patients without *AB.*	Chemotherapy, organ transplant, chronic diseases, invasive procedures, recent bacteremia, tumor, hematological diseases, and recurrent hospitalizations.	P < 0.05
JUNG et al., 2010	South Korea	2008-2009	200	108 patients with bacteremia caused by *AB*.	92 patients without bacteremia.	Respiratory failure, mechanical ventilation, tracheal tube, central venous catheter, bacteremia caused by other microorganisms, previous use of antibiotics.	P < 0.05
NUTMAN et al., 2014	Israel	2008-2011	172	83 patients with bacteremia who died within 14 days.	89 patients with bacteremia who survived after 14 days.	Disease severity and surgical procedure.	P ≤ 0.10
CHUSRI et al., 2015	Thailand	2010-2011	394	139 patients with CR*AB*.	197 patients without *AB* and 58 patients with CS*AB*.	Use of fluoroquinolones, broad spectrum cephalosporins, and carbapenems > 3 days.	P < 0.05
MOGHNIEH et al., 2016	Lebanon	2012-2013	257	40 patients with *AB.*	217 patients without *AB.*	Use of urinary catheter, ICU contact pressure, gastrectomy tube, and carbapenem use.	P < 0.05
GUO et al., 2016	China	2012-2015	87	64 patients with bloodstream infection by MDR*AB*.	23 patients with bloodstream infection by CS*AB*.	Pneumonia, drain use, ICU stay> 7 days, and use of mechanical ventilation.	P < 0.05

***AB*:** *A. baumannii*; **MDR*AB*:** multidrug-resistant *A. baumannii*; **PAVM:** pneumonia associated with mechanical ventilation; **MV:** mechanical ventilation; **CR*AB*:** carbapenem-resistant *A. baumannii*; **CS*AB*:** carbapenem-susceptible *A. baumannii*; **ICU:** intensive care unit.

**TABLE 2:** Risk factors associated with infection and colonization caused by *A. baumannii* in pediatric and neonatal ICUs.

Study	Place of study	Study period	No. of patients	Cases	Controls	Risk factors	P-value
BRITO et al., 2010	Brazil	2001-2002	33	11 patients with infectious conditions caused by *AB.*	22 patients without infectious conditions caused by *AB*.	Birth weight <2500 grams, respiratory syndromes, parental feeding, re-intubation, carbapenem use, and mechanical ventilation.	P < 0.05
DENG et al., 2011	China	2002-2008	349	117 patients with PAVM caused by *AB.*	232 patients without PAVM caused by *AB*.	Use of mechanical ventilation> 7 days.	P < 0.01
HSU et al., 2014	Taiwan	2004-2010	248	37 patients with bacteremia caused by *AB.*	74 patients without bacteremia and 137 patients with bacteremia caused by *Escherichia coli* or *Klebsiella* spp.	Cholestasis, gestational age < 29 weeks.	P < 0.05
LEE et al., 2017	China	2004-2014	40	37 patients with *AB* susceptible to imipenem	3 patients with *AB* resistant to imipenem	Prematurity, low birth weight (70% < 1500 g), prolonged intubation, percutaneous use of central venous catheter, inappropriate initial therapy, infection within the first 10 days of life, use of imipenem for up to 5 days, and high frequency oscillation ventilation.	P < 0.05
PUNPANICH et al., 2012	Thailand	2005-2010	176	91 patients with bacteremia caused by CR*AB*.	85 patients with bacteremia caused by CS*AB*.	Prematurity, use of mechanical ventilation, previous exposure to carbapenems.	P < 0.05
HOSOGLU et al., 2012	Turkey	2006-2007	192	64 patients with *AB* sepsis.	128 patients with blood samples without *AB*.	Stay in the ICU> 7 days, re-intubation.	P < 0.001
De OLIVEIRA COSTA et al., 2015	Brazil	2009-2012	101	47 patients with infection caused by BGN.	54 patients without infection caused by BGN.	Hematologic diseases, neutropenia > 3 days, previous use of antibiotics, previous hospitalization, stay in the ICU > 3 days.	P < 0.05
THATRIMONTRICHAI et al., 2013	Thailand	2009-2014	101	63 patients with CR*AB* pneumonia and 13 patients with CS*AB*.	25 patients with pneumonia without bacterial growth or caused by other microorganisms.	Weight of newborns, previous use of cephalosporins, surfactant replacement therapy, re-intubation, umbilical artery catheterization.	P < 0.05
REDDY et al., 2015	South Africa	2010	388	194 patients with blood culture or respiratory sample positive for *AB*.	194 patients with blood culture or negative respiratory sample for *AB*.	Mechanical ventilation and traumatic brain injury.	P < 0.05
ZARRILLI et al., 2012	Italy	2010-2011	161	22 patients with *AB.*	139 patients without *AB* in the first 48 h.	Use of mechanical ventilation and central venous catheter.	
TRAN et al., 2015	Vietnam	2010-2011	2555	69 patients with sepsis caused by *AB*.	2486 patients without sepsis caused by *AB*.	Maternal infection, gestational age, central catheter, surgical procedure, and blood transfusion.	P < 0.05
KUMAR et al., 2014	India	2010-2012	65	33 patients with CR*AB* bloodstream infection.	32 patients without CS*AB* bloodstream infection.	Previous use of antibiotics, hospitalization > 7 days, use of mechanical ventilation > 7 days.	P < 0.05
WEI et al., 2014	Taiwan	2010-2013	59	12 deaths due to sepsis caused by MDR*AB*.	47 deaths due to sepsis caused by other microorganisms.	Prolonged intubation, mechanical ventilation, peripheral central venous catheter, umbilical catheter, total parental nutrition, ICU stay > 7 days, surgical procedure, and bronchopulmonary dysplasia.	P < 0.05
MACIEL et al., 2017	Brazil	2013-2015	21	21 patients with *AB* colonization without clinical manifestation.	17 patients without sepsis.	Low birth weight, prematurity, hospitalization time, previous exposure to beta-lactams, use of peripheral access, and respiratory syndromes.	P < 0.05

***AB*:** *A. baumannii*; **PAVM:** pneumonia associated with mechanical ventilation; **CR*AB*:** carbapenem-resistant *A. baumannii*; **CS*AB*:** carbapenem-susceptible *A. baumannii*; **BGN:** gram-negative bacillus; **MDR*AB*:** multidrug-resistant *A. baumannii*; **ICU:** intensive care unit.

Skin colonization, length of hospital stays > 7 days, use of corticosteroids, and invasive procedures such as central venous catheter or tracheostomy, were the main risk factors related to the development of pneumonia associated with mechanical ventilation by MDR *A. baumannii* in hospitalized patients (Table 1)^69^ ^,^ ^70^. Risk factors such as use of urinary catheters for more than 6 days, ICU contact pressure > 4 days, presence of gastrectomy tubes, chemotherapy, organ transplantation, chronic diseases, invasive procedures, recent bacteremia, tumors, hematological diseases, recurrent hospitalizations, hospitalization time > 7 days, transfer from another hospital, and previous use of carbapenems or broad-spectrum cephalosporins were related to acquisition of MDR *A. baumannii* infection in adult patients hospitalized in the ICU^69^ ^,^ ^71^. Isolation of MDR *A. baumannii* after medical ICU (MICU) admission was related to a greater likelihood of the patient being older^72^. Previous hospitalization was associated with the isolation of *A. baumannii* after admission to the surgical ICU (SICU). Positive colonization in SICU was strongly correlated with heart failure, paralysis, human immunodeficiency virus infection and acquired immune deficiency syndrome (HIV-AIDS), and rheumatoid arthritis^73^.

Bloodstream infections by *A. baumannii* are frequent in ICUs and have been associated with central venous catheters, mechanical ventilation, pneumonia, drain use, and respiratory and cardiovascular failure^74^. The risk of bacteremia caused by *A. baumannii* was associated with respiratory failure, mechanical ventilation, endotracheal tubes, central venous catheters, surgical procedures, and previous use of antibiotics^75^ ^,^ ^76^.

Newborns are considered susceptible to *A. baumannii* colonization and infections, since they have immature immune systems. The risk is greater for newborns if they are also preterm (< 28 weeks) and underweight (< 2,500 g)^76^ ^,^ ^77^. Birth weight < 2500 grams, respiratory syndromes, parental feeding, re-intubation, carbapenem use, mechanical ventilation, hematologic diseases, neutropenia > 3 days, previous use of broad-spectrum antibiotics, use of invasive devices, immunosuppressants, corticosteroids, previous hospitalization, and ICU stay > 3 days were considered risk factors for the acquisition of *A. baumannii* infections in the neonatal ICU (Table 2)^78^ ^-^ ^80^.

Bloodstream infections caused by *A. baumannii* in neonates were related to the use of mechanical ventilation, and additionally to the presence of traumatic brain injury, previous use of antibiotics, hospitalization > 7 days, and use of mechanical ventilation > 7 days^81^ ^-^ ^83^. The weight of newborns (1000-1499 g), previous use of cephalosporins, surfactant replacement therapy, re-intubation, and umbilical artery catheterization were also indicated as risk factors for the development of neonatal pneumonia caused by carbapenem-resistant *A. baumannii* ^*84*^. Maternal infection, gestational age among 26 to 36 weeks, use of central venous catheters, surgical procedures, blood transfusions, prolonged intubation, use of mechanical ventilation, central peripheral venous catheters, umbilical catheters, total parental nutrition, ICU stay > 7 days, surgical procedures, and bronchopulmonary dysplasia were described as risk factors for sepsis by *A. baumannii* ^77^ ^,^ ^85^. Cholestasis, gestational age < 29 weeks, prematurity, low birth weight (70% < 1500 g), prolonged intubation, central venous catheters, use of imipenem for up to 5 days, mechanical ventilation, and prior carbapenem exposure are related to *A. baumannii* bacteremia in neonates^10^ ^,^ ^86^ ^,^ ^87^. Similar results were reported for colonization in neonates^88^. These studies pinpoint persistent endemic isolates in hospitals, highlighting the need to implement efficient control measures and prevent outbreaks.

 Seasonality of *A. baumannii* infection is another risk factor that should be taken into consideration. A systematic review compiled studies showing 57.1% (12/21) of *A. baumannii* infections occurred in warmer seasons. The hypothesis for this was that it was due to enhanced lipid A moiety regulation, which was responsible for the virulence; it was also reported there was biofilm formation and a higher flow of people entering the hospital facility (carriers, patients, healthcare workers, and sanitation workers) in warmer months. This study highlights the importance of correlating different factors of *A. baumannii* adaptability in the ambient environment to implement preventive measures for seasonal peaks of infection^89^.

Information related to colonization pressure (CP) is important for mediating risk factors. CP is a tool to measure the proportion of *A. baumannii* reservoirs within a health care facility. For *A. baumannii* surveillance, CP can help enhance patient screening and determine infection control measures^90^ ^,^ ^91^.

## MOLECULAR EPIDEMIOLOGY OF *A. BAUMANNII* IN BRAZIL

In Brazil, the first outbreak associated with OXA-23-producing *A. baumannii* isolates was in 1999^92^. Subsequently, different outbreaks were reported^93^. *A. baumannii* dissemination in different Brazilian hospitals was associated with *bla*_OXA-51_ and *bla*_OXA-23_ genes and highlighted the prevalence of *ISAba1*/OXA-23 and *ISAba1*/OXA-51 genetic profiles^94^. Isolates carrying the *bla*_OXA-51_, *bla*_OXA-58_, and *bla*_OXA-23_ genes, and *ISAba1* upstream of OXA-51 and OXA-23 were found in different ICUs, indicating an outbreak of cross-contamination among patients, equipment, or medical staff^94^. The *bla*_OXA-58_ and *bla*_OXA-65_ genes with the upstream *ISAba1* sequence for both genes have been reported. The *bla*_OXA-58_ gene is prevalent in Argentina, indicating a possible spread from the border with Rio Grande do Sul^95^. In addition, two genotypes of OXA-23-producing *A. baumannii* were present at 8 hospitals in the same city, suggesting the spread of isolates in these environments^93^. The sequence type (ST) 156, ST25, and ST160 were identified in a Brazilian hospital^96^. Cephalosporin-resistant *A. baumannii* and producers of extended-spectrum beta-lactamases (ESBL) were identified in a neonatal intensive care unit (NICU), causing septicemia in hospitalized neonates (Table 3)^5^. A study in neonates described most isolates as belonging to ST1 and had *ISAba1* upstream of the *bla*_OXA-51_ and *bla*_OXA-23_ genes^88^.

**TABLE 3:** Outbreaks of *Acinetobacter baumannii* in Brazil.

Study	Place of study	Year of outbreak	Place of outbreak	No. of patients	Antibiotic Resistance	Reported genes
DALIA-COSTA et al., 2003	Curitiba	1999	Ward	8	IPM, MEM, CIP, and AMG	*bla*_OXA-23_
BRITO et al., 2005	Uberlândia	2005	NICU	11	GEN, CIP, CAZ, FEP, and ATM	-
TAKAGI et al., 2009	São Paulo	2005-2006	ICU	8	PIP, TZP, CAZ, CTX, ATM, IPM, MEM, CIP, AMK, GEN, and SXT	*bla*_OXA-51_
MARTINS et al., 2009	Porto Alegre	2007	DHW	53	CIP, GEN, TZP, and SXT	*bla*_OXA-51,_ *bla*_OXA-23_
GUSATTI et al., 2012	Porto Alegre	2007	Ward	74	IPM, MEM, AMK, CIP, GEN, CET, AMA, SXT, and TIM	*bla*_OXA-51,_ *bla*_OXA-58,_ *bla*_OXA-65,_ *ISAba1*/OXA-51
PAGANO et al., 2015	Porto Alegre	2011	DHW	122	FEP, CIP, CAZ, AMA, AMK, PMB, IMP and MEM	*bla*_OXA-23_
CASTILHO et al., 2017	Goiás	2010	ICU	64	AMA, FEP, AMK, PMB, and TGC	*ISAba1*/OXA-23 and *ISAba1*/OXA-51, *bla*_OXA-51,_ *bla*_OXA-23,_ *bla*_OXA-58_
MACIEL et al., 2017	Dourados	2013-2015	NICU	21	AMA, TZP, CAZ, CRO, FEP, GEN, AMK, CIP, and TGC.	*ISAba1*/OXA-23 and *ISAba1*/OXA-51

**ICU:** intensive care unit; **NICU:** neonatal intensive care unit; **DHW:** different hospital wards; **IPM:** imipenem; **MEM:** meropenem; **CIP:** ciprofloxacin; **AMG:** aminoglycoside; **GEN:** gentamicin; **CAZ:** ceftazidime; **FEP:** cefepime; **ATM:** aztreonam; **TZP:** piperacillin/tazobactam; **AMK:** amikacin; **SXT:** trimethoprim/sulfamethoxazole; **CET:** cephalothin; **TIM:** ticarcillin/clavulanic acid; **PMB:** polymyxin B; **TGC:** tigecycline; **CRO:** ceftriaxone; **AMA:** ampicillin/sulbactam; **CTX:** cefotaxime; **PIP:** piperacillin.

A study in Recife, Brazil described isolates belonging to ST1, ST15, ST25, ST79, ST113, and ST881 (related to ST1). Among them, ST79 and ST113 were found to be more virulent and presented resistance genes. ST113 and ST15 were commonly found in all 5 hospitals of the study, while ST79 was found in 4 hospitals and ST1 in 3 hospitals. Among the CCs circulating between hospitals, Leal et al. described CC1, CC15, and CC113, which are globally spread types, and CC79, which is found in South America, North America, and Europe^97^.

A study carried out in nine hospitals in South America identified *A. baumannii* clinical isolates presenting *bla*_OXA-51_, *bla*_OXA-23_, *bla*_OXA-72_, *bla*_OXA-132_, *bla*_OXA-65_, *bla*_OXA-69_, and *bla*_OXA-64_ genes. Multilocus sequence type (MLST) analysis identified ST79, ST25, and ST15^98^. The two major clonal complexes (CC) found in *bla*_OXA-23_ multidrug-resistant *A. baumannii* are CC15 and CC79, and CC15 has already been described in 9 Brazilian states^77^. In addition, ST15 was described in other countries, such as Argentina and Turkey, and ST79 was described in the United States, Canada, and Spain^99^. Of the clonal profiles identified, ST15 and ST79 were described in several countries, indicating their spread among hospitals around the world and high mortality rates^100^.

**FIGURE 1:**
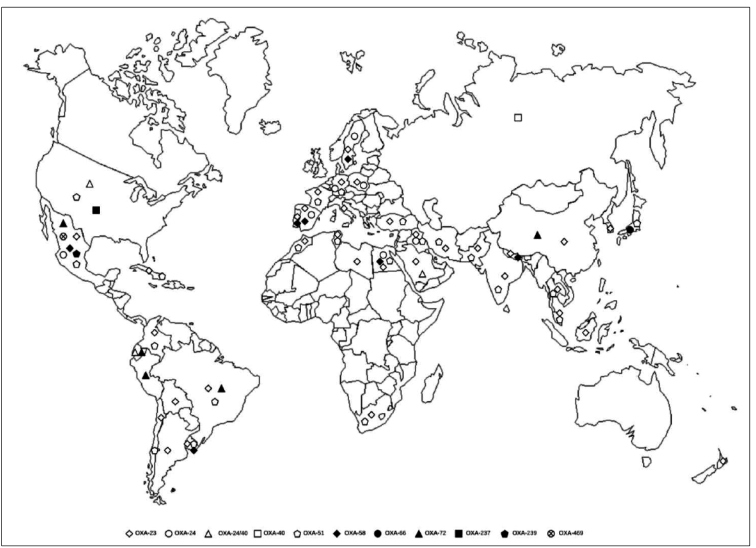
Geographic distribution of OXA enzymes in the last seven years.

The Antimicrobial Surveillance Program (SENTRY) evaluated the prevalence of *Acinetobacter* spp. and other gram-negative bacilli isolated from Latin American (Argentina, Brazil, Chile, and Mexico) medical centers from 2008 to 2010. In this period, 5,704 gram-negative bacilli were isolated and 845 (17.7%) were classified as *Acinetobacter* spp. This microorganism was responsible for 7.2% of the 6,035 bloodstream infections, 7% of the 1,442 pneumonia cases, and 9.9% of the 1,531 skin and soft tissue infections. The oxacillinases found in this study were OXA-23 and OXA-24 in Argentina, OXA-23 in Brazil, OXA-58 in Chile, and OXA-24 in Mexico^101^. Figure 1 shows a map representing the description of the resistant gene OXA in the last eight years^3^ ^,^ ^102^ ^-^ ^145^.

## MOLECULAR EPIDEMIOLOGY OF *A. BAUMANNII* IN THE WORLD

In France, 110 *A. baumannii* clinical strains were isolated between 2010 and 2011. Of these, 90 isolates harbored *bla*_OXA-23_, 12 *bla*_OXA-24_, and 8 *bla*_OXA-58_. One of the isolates simultaneously displayed *bla*_OXA-23_ and *bla*_PER-1_, and 2 isolates possessed *bla*_OXA-23_ and *bla*_OXA-58_. Pulsed-field gel electrophoresis (PFGE) analysis showed 30 clusters and MLST revealed 11 STs (ST115, ST1, ST2, ST10, ST20, ST25, ST79, ST85, ST107, ST108, and ST125)^43^. A study conducted in China evaluated 57 clinical isolates of carbapenem-resistant *A. baumannii* that were positive for the *bla*_OXA-23_/*ISAba1* and *bla*_OXA-51_ genes, harboring ST75 and ST137^145^. In addition, a Chinese hospital identified transposons Tn2006, Tn2007, and Tn2008 in 59 clinical isolates of OXA-23-producing *A. baumannii* ^146^.

In Saudi Arabia, 107 *A. baumannii* clinical isolates were identified, of which 75 harbored the genes *bla*_TEM_ and *bla*_CTX-M_ (n = 86), *bla*_OXA-51_ (n = 100)*, and bla*_OXA-23_ (n = 97). MLST analysis identified ST195, ST557, ST208, ST499, ST218, ST231, ST222, and ST286, all belonging to CC2, except ST231^147^. In the United States, in 2008 and 2009, 65 *A. baumannii* clinical isolates producing *bla*_OXA-51_/*ISAba1* were found in different hospitals, harboring *bla*_OXA-23_ (65/65) and *bla*_OXA-40_ genes (09/65). PFGE analysis indicated 24 clusters, whereas MLST identified ST1, ST2, ST77, ST79, ST123, ST124, CC1, and CC2^148^. A total of 149 clinical isolates of *A. baumannii*, containing *bla*_OXA-58_ (n = 31), *bla*_OXA-58_/*ISAba3* (n = 14), and *bla*_OXA-72_ (n = 18) were isolated from different hospitals in Egypt. These presented as 54 clusters by PFGE and ST763, ST777, ST369, ST762, and ST229 were identified^149^.

In South Africa, 94 clinical isolates of *A. baumannii* were found in different hospitals; 93 carried the *bla*_OXA-51_ gene and 72 the *bla*_OXA-23_. PFGE analysis grouped the isolates into 4 clusters with 5 STs (ST106, ST258, ST339, ST502, ST758, ST848), in which ST258 and ST758 corresponded to the international clone I, and ST502 and ST848 to the international clone II^150^. In India, 100 *A. baumannii* strains showed high genetic variability. MLST identified ST110, ST108, ST194, ST14, ST146, ST69, ST188, ST386, ST387, ST388, ST389, ST390, and ST391^151^. A total of 160 *A. baumannii* clinical isolates were identified in Vietnam, of which 119 were MDR or extensively resistant, presenting a high level of resistance against third- and fourth-generation cephalosporins. Of these, 128 isolates harbored the *bla*_OXA-51_ and *bla*_OXA-23_ genes associated with the *ISAba1* element. MLST analysis identified 16 STs from 23 isolates, confirmed new STs, and some isolates belonged to ST136^152^.

In Malaysia, 162 clinical isolates of MDR *A. baumannii* were identified, of which 128 were resistant to carbapenems. The *bla*_OXA-23_, *bla*_OXA-IMP,_ and *bla*_OXA-ADC_ genes were identified, and *ISAba1,* upstream of the *bla*_OXA-23_ and *bla*_OXA-ADC_ genes, was also found. Point mutations in *gyrA* (Ser83Leu) and *parC* (Ser80Leu), which provide resistance to ciprofloxacin, were also identified in the isolates. MLST identified two predominant STs (ST195 and ST208)^104^.

Molecular typing of *A. baumannii* provides a better understanding of the epidemiology of outbreaks and identification of cross-transmission, as well as assisting in the monitoring and control of nosocomial infections^17^ ^,^ ^153^. Thus, several methods have been used to study the molecular epidemiology of *A. baumannii* and analyze the mechanisms involved in the resistance of this microorganism.

## CONCLUSION

The increase in healthcare-associated infection (HAI) rates connected to *A. baumannii* antimicrobial resistance has become a major public health challenge worldwide. *A. baumannii* possesses several resistance mechanisms. However, hydrolysis by OXA-type carbapenemases and metallo-β-lactamases are considered the most prevalent mechanisms conferring resistance to most beta-lactam antibiotics and reduce therapeutic options. This study highlights the occurrence of outbreaks in hospital settings, especially in ICUs, which are commonly related to prolonged hospital stays and invasive procedures. Thus, epidemiological studies are important for monitoring the occurrence of *A. baumannii* clinical isolates and may assist in the implementation of appropriate measures, contributing to the control of hospital infections.
